# Sarcopenia Versus Systemic Inflammation as Predictors of New Vertebral Fractures After Vertebroplasty or Kyphoplasty: A Retrospective Cohort Study

**DOI:** 10.3390/jcm15103677

**Published:** 2026-05-11

**Authors:** Ali Maksut Aykut, Mustafa Emrah Kaya, Yurdal Serarslan, Atilla Yilmaz, Mustafa Aras

**Affiliations:** 1 Department of Neurosurgery, Mustafa Kemal University, Faculty of Medicine Hospital, 31060 Antakya, Turkey; dralimaksut@gmail.com; 2 Department of Neurosurgery, Ordu University, Faculty of Medicine Hospital, 52200 Ordu, Turkey; yserarslan@yahoo.com; 3 Department of Neurosurgery, Medicana Hospital, 34520 Istanbul, Turkey; atillayilmaz@hotmail.com; 4 Department of Neurosurgery, Ondokuz Mayıs University, Faculty of Medicine Hospital, 55139 Samsun, Turkey; mustafa.aras@omu.edu.tr

**Keywords:** osteoporotic vertebral compression fracture, vertebroplasty, kyphoplasty, sarcopenia, Psoas muscle, systemic inflammation, NLR, SII, new fracture prediction

## Abstract

**Background**: Osteoporotic vertebral compression fractures (OVCFs) are among the most 11 common fragility fractures in the elderly. Although vertebroplasty and kyphoplasty provide effective pain relief, new vertebral fractures remain a significant concern postoperatively. Imaging parameters associated with sarcopenia and systemic inflammatory markers have been individually associated with fracture risk, but their combined predictive value in the postoperative period has not been adequately defined. **Methods**: This retrospective cohort study included 166 patients who underwent vertebroplasty or kyphoplasty for OVCFs with a follow-up period of at least 12 months. Cross-sectional area (CSA) and density (HU) of the Psoas muscle were measured at the L3 mid vertebral level on preoperative CT. Preoperative hematological indices (NLR, PLR, LMR, SII, lymphocyte count, hemoglobin, and MPV) were recorded. The primary outcome was the development of a new vertebral fracture. Group comparisons were performed using Mann–Whitney U tests with Benjamini–Hochberg correction. Logistic regression identified independent predictors. Internal validation was performed using bootstrap optimism correction (1000 iterations) and 10-fold cross-validation. Calibration was assessed using the Hosmer–Lemeshow test and calibration plots. **Results**: Forty-nine patients (29.5%) developed a new fracture. After multiple comparison correction, Psoas 25 HU (BH-adj *p* < 0.001, r_rb = −0.810), Psoas CSA (BH-adj *p* < 0.001, r_rb = −0.622), NLR (BH-adj *p* = 0.016), lymphocyte count (BH-adj *p* = 0.009), and hemoglobin (BH-adj *p* = 0.033) showed significant differences between groups. SII did not remain significant after multiple-comparison correction (BH-adjusted *p* = 0.092). In multivariate logistic regression, only Psoas CSA (OR = 0.403, 95% CI 0.230–0.708, *p* = 0.002) and Psoas HU (OR = 0.825, 95% CI 0.770–0.885, *p* < 0.001) remained independently significant. The parsimonious model, with adequate calibration (Hosmer–Lemeshow *p* = 0.524), achieved an optimism-adjusted AUC of 0.918 (10-fold CV AUC = 0.924). A Psoas HU threshold of 20.50 yielded 79.6% sensitivity and 94.9% specificity. **Conclusions**: CT-derived Psoas muscle mass and quality are strongly associated with new vertebral fractures after percutaneous vertebral augmentation procedures in this retrospective cohort and showed stronger independent predictive performance than systemic inflammatory markers. These readily accessible imaging biomarkers can aid in risk stratification, although the proposed threshold requires externally validation before clinical implementation.

## 1. Introduction

Osteoporotic vertebral compression fractures (OVCFs) are the most common fragility fractures worldwide, affecting an estimated 1.4 million people annually and representing a significant burden of pain, disability, and mortality in the aging population [1,2]. Percutaneous vertebroplasty (PVP) and kyphoplasty (PKP) have become established minimally invasive treatments, providing rapid pain relief and improved function [3,4]. However, the development of new vertebral compression fractures (NVCFs) after these procedures remains a significant clinical concern, with reported incidence rates varying between 6% and 52% across studies [5,6,7].

Several risk factors have been identified for vertebral fractures following vertebral augmentation surgery. These include advanced age, low bone mineral density, intradiscal cement leakage, and low vertebral CT Hounsfield units [2,8,9]. However, the contribution of parameters related to sarcopenia has received relatively less attention. Sarcopenia, defined as progressive age-related loss in skeletal muscle mass, strength, and quality [10], shares pathophysiological pathways with osteoporosis and is increasingly recognized as an independent contributing factor to fracture risk [11,12]. CT-based measurements of cross-sectional area (CSA) and density (Hounsfield Unit, HU) of the Psoas muscle at the L3 vertebral level provide readily accessible and reproducible sarcopenia markers obtainable from routine preoperative imaging [13,14,15].

Simultaneously, systemic inflammation has emerged as a modifiable factor linking bone metabolism and muscle quality. Hematological inflammatory indices, including neutrophil–lymphocyte ratio (NLR), platelet–lymphocyte ratio (PLR), lymphocyte–monocyte ratio (LMR), and systemic immune-inflammatory index (SII), are inexpensive and widely accessible biomarkers associated with osteoporosis and fracture risk in large epidemiological studies [16,17,18,19]. A recent analysis by the UK Biobank on over 500,000 individuals showed that high NLR, PLR, and SII values independently predicted osteoporosis and fracture occurrence [16]. However, it has not been investigated whether these inflammatory indices retain their independent predictive value after accounting for sarcopenia parameters in the context of new vertebral fractures following vertebral augmentation surgery. This study aimed to evaluate the combined and relative predictive value of CT-derived sarcopenia parameters (Psoas CSA and Psoas HU) and preoperative systemic inflammatory markers for the development of new vertebral fractures after vertebroplasty or kyphoplasty in patients with spinal fractures. We hypothesized that imaging biomarkers related to sarcopenia would show a stronger independent association than hematological inflammatory indices.

## 2. Materials and Methods

### 2.1. Study Design and Population

This retrospective cohort study was conducted at Hatay Mustafa Kemal University Hospital. Consecutive patients who underwent PVP or PKP for OVCFs between January 2018 and December 2025 were identified from the hospital information system. The study protocol was approved by the Institutional Ethics Committee of Hatay Mustafa Kemal University (approval number: Decision No. 44, dated 25 March 2026).

Inclusion criteria were as follows: (1) primary OVCF treated with PVP or PKP; (2) availability of adequate preoperative lumbar CT images for Psoas muscle measurement; (3) preoperative laboratory results obtained within one week before the procedure; and (4) at least 12 months of clinical and radiological follow-up. Exclusion criteria were as follows: (1) vertebral fractures due to trauma, malignancy, or infection; (2) previous vertebral surgery; (3) incomplete follow-up data; and (4) inadequate CT image quality for reliable muscle measurement.

### 2.2. Data Collection

Demographic data (age, gender), clinical data (preoperative Visual Analog Scale [VAS], 6-month VAS, follow-up period), and laboratory parameters (white blood cell count, hemoglobin, hematocrit, platelet count, mean platelet volume [MPV], neutrophil count, lymphocyte count, monocyte count) were obtained from electronic medical records. Composite inflammation indices were calculated: NLR (neutrophil/lymphocyte), PLR (platelet/lymphocyte), LMR (lymphocyte/monocyte), and SII (platelet × neutrophil/lymphocyte). Delta VAS was calculated as preoperative VAS minus 6-month VAS; positive values indicate worsening pain, and negative values indicate improvement.

### 2.3. Radiological Measurements

**Figure 1:**
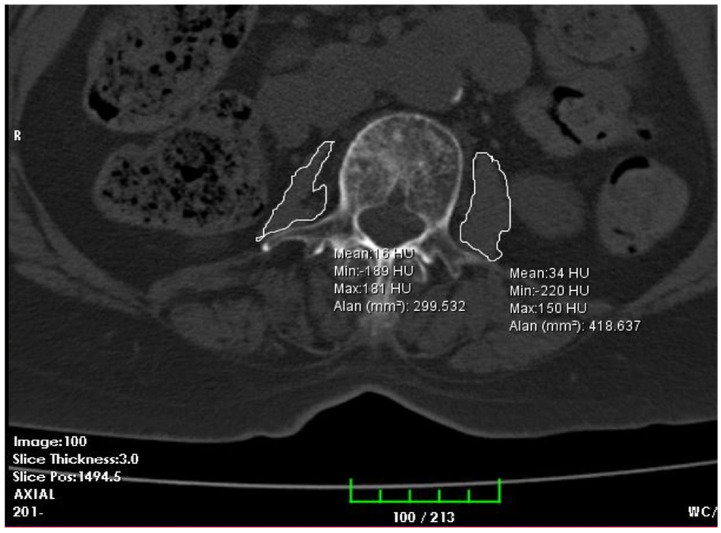
CT-based Psoas muscle measurement. Representative axial preoperative CT image at the mid-level of the L3 vertebra showing bilateral manual region of interest (ROI) delineation of the Psoas muscles. Manual boundary tracing was used to measure cross-sectional area (CSA, mm^2^) and mean muscle density (Hounsfield Units, HU) for each side, and the arithmetic mean of bilateral measurements was used for the analysis. Slice thickness: 3.0 mm.

Psoas muscle measurements were performed by two independent surgeons at the mid-L3 vertebral level on preoperative axial CT images. The cross-sectional area (cm^2^) of the Psoas muscle was measured bilaterally using a manual method of defining muscle boundaries; the arithmetic mean of the right and left sides was recorded. Psoas muscle density (HU) was automatically measured within defined regions, and the bilateral arithmetic mean was used. A representative axial CT image showing the delineation of the Psoas muscle region of interest at the mid-L3 level for CSA and HU measurements, which are included in Figure 1. Because the available dataset contains mean measurements rather than individual observer-specific values, a formal retrospective inter-observer and intra-observer reliability analysis could not be performed. The presence of new vertebral fractures during the follow-up period was determined on plain radiographs and/or CT images obtained during clinical visits. New fractures were classified as adjacent (one level above or below the treated vertebra) or distant (two or more levels away from the treated vertebra).

### 2.4. Outcome Definition

The primary outcome was the development of any new vertebral fracture assessed radiologically during the follow-up period and coded as a binary variable (present/absent).

### 2.5. Statistical Analysis

All continuous variables were assessed for normality using the Shapiro–Wilk test. Since most variables did not exhibit normal distribution, group comparisons fracture and no-fracture groups were performed using Mann–Whitney U tests for continuous variables and chi-square tests for categorical variables. Benjamini–Hochberg false discovery rate (FDR) correction was applied to all 17 univariate comparisons to account for multiple testing. Effect sizes were quantified using rank-biserial correlation (r_rb) and Hodges–Lehmann median difference. Univariate logistic regression was performed for each candidate predictor. Variables with *p* < 0.10 in univariate analysis were considered for multivariate modeling. Age and sex were retained in the parsimonious model because of their established roles as clinically relevant confounders in fracture risk, despite the moderate number of events. Model selection prioritized parsimony and avoidance of overfitting. Multicollinearity was assessed using variance inflation factors (VIF), with VIF > 5 indicating problematic collinearity. Model selection was guided by the Akaike Information Criterion (AIC), Bayesian Information Criterion (BIC), number of events per variable (EPV), and discrimination (AUC). The adequacy threshold was set as EPV ≥ 10.

Internal validation of the final model was performed using (1) bootstrap optimism correction with 1000 resampling iterations and (2) stratified 10-fold cross-validation. Model calibration was visually assessed with calibration plots using the Hosmer–Lemeshow goodness-of-fit test and LOWESS smoother. Receiver operating characteristic (ROC) curves were generated for all significant predictors; AUCs were reported with 95% bootstrap confidence intervals (1000 iterations) and optimal cutoff points (Youden’s J index). Spearman rank correlations examined the relationships between underlying variables. All analyses were performed using Python 3.12 (SciPy, statsmodels, scikit-learn). Two-tailed *p* < 0.05 was considered statistically significant, and FDR correction was applied where indicated.

## 3. Results

### 3.1. Patient Characteristics

A total of 166 patients met the inclusion criteria. The cohort was predominantly female (*n* = 126, 76.0%), with a median age of 68 years (IQR 60–76). The median follow-up period was 12 months (range 12–24). Forty-nine patients (29.5%) developed new vertebral fractures during the follow-up period: 23 (46.9%) in adjacent segments and 26 (53.1%) in distant segments.

### 3.2. Univariate Group Comparisons

Table 1 presents a comparison of all parameters between fracture and non-fracture groups. After Benjamini–Hochberg correction, eight of the 17 variables retained their significance at FDR < 0.05. The largest effect sizes were observed for Psoas HU (r_rb = −0.810, Hodges–Lehmann Δ = −16.5 HU) and Psoas CSA (r_rb = −0.622, HLΔ = −1.66 cm^2^). Patients with newly developed fractures showed significantly lower Psoas HU (median 17.50 vs. 34.00, *p* < 0.001), lower Psoas CSA (3.95 vs. 5.43 cm^2^, *p* < 0.001), higher NLR (3.28 vs. 2.35, BH-adj *p* = 0.016), lower lymphocyte count (1.51 vs. 1.95, BH-adj *p* = 0.009), and lower hemoglobin (11.90 vs. 12.50 g/dL, BH-adj *p* = 0.033). Notably, SII did not maintain its significance after multiple comparison correction (raw *p* = 0.049, BH-adjusted *p* = 0.092). Delta VAS was strongly positive in the fracture group (median +3) compared to the non-fracture group (median −8), indicating worsening pain in those with new fractures (*p* < 0.001). Gender distribution approached significance but did not reach significance (chi-square *p* = 0.087).

**Figure 2:**
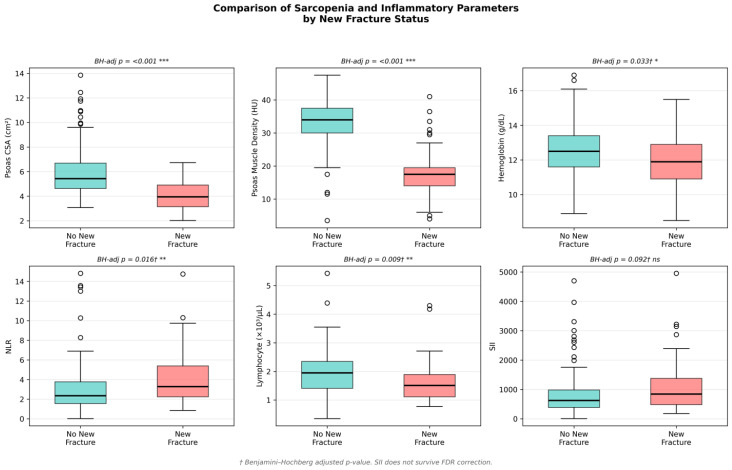
Distribution of baseline sarcopenia and inflammation parameters according to recent spinal fracture status. Box plots show the distribution of Psoas muscle density (HU), Psoas cross-sectional area (CSA), neutrophil–lymphocyte ratio (NLR), and lymphocyte count in patients with and without recent spinal fracture. Midlines represent medians, boxes represent interquartile ranges (IQR), and whiskers represent the range within 1.5 × IQR. Outliers are shown as separate points. ns, *, ** and *** in the table indicate not significant, *p* < 0.05, *p* < 0.01 and *p* < 0.001, respectively.

The distributions of key sarcopenia and inflammation parameters by fracture status are illustrated in Figure 2.

### 3.3. Univariate Logistic Regression

Table 2 presents the results of the univariate logistic regression. Delta VAS was excluded as a postoperative outcome rather than a primary predictor. Psoas HU showed the strongest univariate association (OR = 0.810 per 1 HU increase, 95% CI 0.761–0.862, *p* < 0.001), followed by Psoas CSA (OR = 0.336 per 1 cm^2^ increase, 95% CI 0.222–0.508, *p* < 0.001). Among inflammation markers, lymphocyte count (OR = 0.537, *p* = 0.020), hemoglobin (OR = 0.749, *p* = 0.010), and NLR (OR = 1.122, *p* = 0.063) showed significant correlations.

### 3.4. Multivariate Logistic Regression and Model Selection

In the full 7-predictor model (Model A), VIF analysis revealed problematic multicollinearity for NLR (VIF = 6.07) and SII (VIF = 5.97), consistent with their common hematological components. Based on the lowest AIC (106.9) and BIC (122.5), adequate EPV (12.2), and all VIFs being below 2.3, a parsimonious model (Model C: age, sex, Psoas CSA, Psoas HU) with negligible discrimination loss (AUC 0.927, compared to 0.927 for the full model) was selected. In this parsimonious model (Table 3), only Psoas CSA (OR = 0.403, 95% CI 0.230–0.708, *p* = 0.002) and Psoas HU (OR = 0.825, 95% CI 0.770–0.885, *p* < 0.001) were independently significant. Each 1 cm^2^ increase in Psoas CSA reduced the odds of a new fracture by approximately 60%, while each 1 HU increase in muscle density reduced it by approximately 18%. Systemic inflammatory markers did not provide additional independent predictive value beyond sarcopenia parameters.

### 3.5. Internal Validation

Bootstrap optimism correction (1000 iterations) yielded an average optimism value of 0.010, which corresponded to an optimism-adjusted AUC of 0.918. Stratified 10-fold cross-validation confirmed robust out-of-sample discrimination: CV-AUC 0.924 (SD = 0.075). The Hosmer–Lemeshow test showed sufficient calibration (χ^2^ = 7.12, df = 8, *p* = 0.524). The calibration plot demonstrated that predicted probabilities closely tracked observed proportions across deciles, indicating no systematic miscalibration (Supplementary Figure S2). The Hosmer–Lemeshow test was supplemented by visual assessment, as the test may be dependent on sample size.

### 3.6. ROC Analysis

Table 4 presents the ROC analysis for the individual predictors. Psoas HU showed the highest univariate discriminatory power (AUC = 0.905, 95% CI 0.844–0.955), with a sample-derived exploratory cutoff point of 20.50 HU (sensitivity 79.6%, specificity 94.9%). Psoas CSA achieved an AUC of 0.811 (cutoff point 4.18 cm^2^). Inflammatory markers showed moderate discrimination (AUC range 0.597–0.651). The combined Psoas CSA + HU logistic model achieved an apparent AUC of 0.920 and an optimism-adjusted AUC of 0.916.

**Figure 3:**
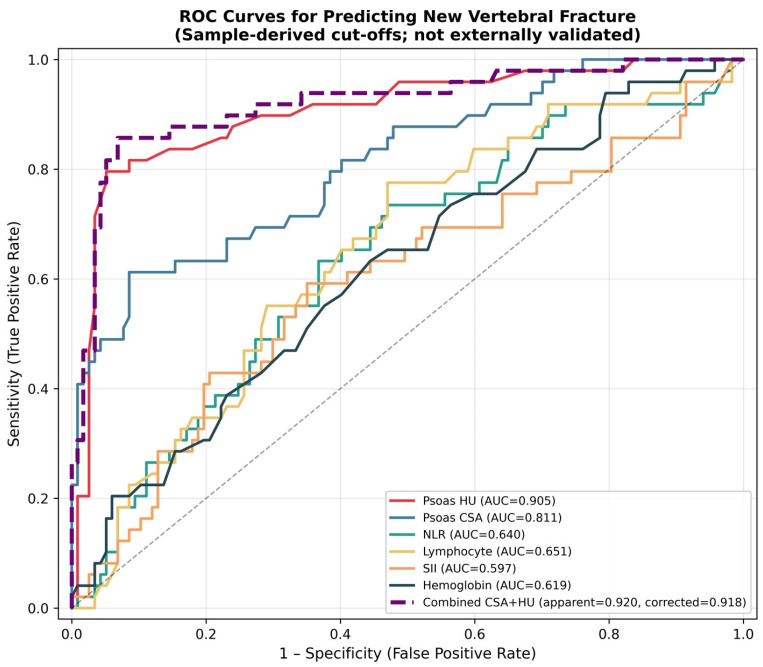
ROC curves for predicting new vertebral fracture. The combined CSA + HU model shows the apparent and optimism-adjusted AUC. All threshold values are derived from the sample. The dashed diagonal line indicates the reference line for no discrimination (AUC = 0.50).

Figure 3 displays the ROC curves for all individual predictors and the combined CSA + HU model, along with both apparent and optimism-corrected AUC values.

### 3.7. Exploratory Subgroup Analysis

Among 49 patients with new fractures, those with distant fractures were significantly older than those with adjacent fractures (median 74.5 vs. 67.0 years, *p* = 0.045). No significant differences were observed between adjacent and distant subgroups in Psoas CSA, Psoas HU, inflammatory markers, or Delta VAS; this is consistent with a systemic rather than a local mechanical risk mechanism; however, these subgroup comparisons are exploratory due to small sample sizes (*n* = 23 vs. *n* = 26).

### 3.8. Correlation Analysis

**Figure 4:**
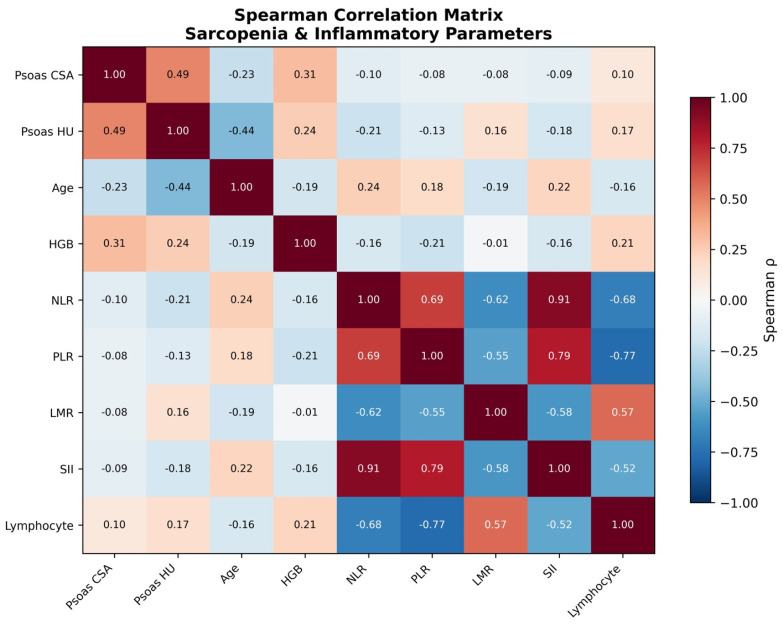
Spearman correlation matrix. Note the NLR–SII collinearity (ρ = 0.91).

Spearman correlations revealed a moderately positive correlation between Psoas CSA and Psoas HU (ρ = 0.488, *p* < 0.001), confirming that they measure related but non-redundant constructs. Psoas HU showed a significant inverse correlation with NLR (ρ = −0.214, *p* = 0.006), consistent with the relationship between systemic inflammation and impaired muscle quality. Both Psoas measurements showed a positive correlation with hemoglobin (CSA: ρ = 0.311; HU: ρ = 0.245) and an inverse correlation with age (CSA: ρ = −0.230; HU: ρ = −0.439). NLR and SII showed a high degree of linear correlation (ρ = 0.907), supporting the decision not to include either in multivariate models. The full Spearman correlation matrix of sarcopenia and inflammation parameters is presented in Figure 4.

## 4. Discussion

This study reveals that Psoas muscle mass (CSA) and muscle quality (HU) obtained by CT are strong and independent predictors of new vertebral fractures after vertebroplasty or kyphoplasty for OVCFs, significantly outperforming systemic inflammatory markers. To our knowledge, there are few studies that have evaluated sarcopenia parameters obtained by CT and a comprehensive hematological inflammatory index panel, along with rigorous internal validation and multiple comparison control, as predictors of new fractures after augmentation.

### 4.1. Sarcopenia as the Dominant Determinant

The central finding—that Psoas HU and Psoas CSA were the only independent predictors in the multivariate analysis—aligns with and expands upon previous evidence linking sarcopenia to osteoporotic fracture risk. Jiang et al. [20] recently supported the concept of “osteosarcopenia” as a composite risk phenotype by showing that the cross-sectional area (CSA) of the Psoas muscle at L3 was significantly lower in patients at risk of vertebral fracture following OVCF. Similarly, Lidar et al. [21] reported that normalized Psoas CSA was an independent risk factor for subsequent fractures after percutaneous vertebral augmentation in elderly patients. Importantly, our study includes the dimension of muscle quality (density in HU) in addition to muscle quantity (CSA). The significantly superior discriminatory power of Psoas HU (AUC = 0.905) compared to Psoas CSA (AUC = 0.811) suggests that fat infiltration of the Psoas muscle (reflected by lower HU values) may be a more informative indicator of fracture susceptibility than muscle size alone. This is consistent with the concept known as myosteatosis, where intramuscular fat accumulation compromises both the mechanical support and endocrine functions of skeletal muscle [11]. The Psoas HU threshold value of 20.50 obtained from the sample in this study is lower than the mean values reported in healthy populations and indicates a significantly degenerated muscle tissue state in the fracture group; however, prospective validation of this threshold value is needed.

### 4.2. Systemic Inflammatory Markers: Significant but Not Independent

Our univariate findings confirm that high NLR and low lymphocyte count are associated with a new fracture risk after vertebral augmentation, consistent with increasing evidence showing a link between chronic low-grade inflammation and impaired bone metabolism [16,17]. In a meta-analysis of eight studies involving 3769 osteoporotic patients, Xu et al. [22] showed that NLR and SII were significantly associated with fracture risk. Fang et al. [19] identified SII as a novel predictor of postmenopausal osteoporotic fracture risk and showed that a cutoff point of 834.89 had discriminative ability.

However, a key finding of our study is that these inflammatory markers lost their statistical significance after adjusting for sarcopenia parameters. This is consistent with the hypothesis that sarcopenia may be a closer predictor of fracture risk in this population and potentially mediate part of the inflammation–fracture pathway. The significant inverse correlation we observed between Psoas HU and NLR (ρ = −0.214, *p* = 0.006) supports this interpretation and suggests that systemic inflammation may contribute to impaired muscle quality, which in turn may compromise spinal stability. However, based on this observational design, we cannot determine whether the lack of significance of inflammatory markers reflects a true lack of contribution, mediation via sarcopenia, or insufficient statistical power to detect independent effects. Further studies, including formal mediation analysis, are needed to clarify the relationship between systemic inflammation, sarcopenia, and fracture risk.

### 4.3. Adjacent and Distant Fractures

It is noteworthy that there were no significant differences in sarcopenia or inflammatory parameters between adjacent and distant new fracture subgroups. Previous studies have proposed different mechanisms for these two types of fractures; adjacent fractures have been attributed partly to altered biomechanics resulting from cement reinforcement, while distant fractures have been linked to the natural progression of osteoporosis [5]. Our exploratory finding that sarcopenia parameters did not differ between subgroups supports the interpretation that muscle impairment functions as a systemic risk factor rather than a local risk factor, although small subgroup sizes limit definitive conclusions.

### 4.4. Clinical Implications

The combined Psoas CSA + HU model achieved an excellent optimism-adjusted AUC of 0.918, and—importantly—these measurements can be opportunistically obtained from preoperative CT scans already performed for procedure planning. This positions Psoas muscle assessment as a practical, cost-effective risk stratification tool. Patients identified as high-risk based on exploratory thresholds such as Psoas HU ≤ 20.50 may be considered for closer follow-up and individualized preventive strategies; however, this threshold should not be considered clinically definitive until externally validated.

### 4.5. Methodological Strengths

Several methodological features strengthen the reliability of our findings. First, Benjamini–Hochberg FDR correction was applied to all univariate comparisons, and SII was appropriately downgraded to exploratory status after failing to survive correction. Second, multicollinearity was formally assessed with VIF analysis, leading to a principled model selection strategy. Third, the final model was internally validated using both bootstrap optimism correction and cross-validation with consistent results (apparent 0.927, corrected 0.918, CV 0.924). Fourth, calibration was assessed with both the Hosmer–Lemeshow test and visual calibration charts. Fifth, the parsimonious model achieved adequate EPV (12.2), and the regression equation was provided for potential replication. Although the initial number of candidate predictors was relatively high for 49 events, the final model was deliberately reduced to a four-variable, simplified structure, met the EPV ≥ 10 criterion, and demonstrated stable performance on bootstrap optimism correction and 10-fold cross-validation.

### 4.6. Limitations

Several limitations must be acknowledged. First, this is a retrospective, single-center study, limiting causal inference and external generalizability. Therefore, the findings should be interpreted as observed relationships within this cohort, not as externally validated clinical predictive rules. Second, although EPV was adequate (12.2), the moderate sample size and (49 events) limited the number of predictors that could be included and reduced the sensitivity of subgroup analyses. Third, all cutoff values were derived from the sample using Youden’s J-index and were not externally validated; therefore, they should be considered exploratory and hypothesis-generating. Fourth, BMD and T-score data were missing or unavailable for the analytical cohort, preventing adjustment for osteoporosis severity. This is a significant limitation because osteoporosis severity is a major predictor of fracture risk and may act as a residual confounding factor. Fifth, formal inter-observer and intra-observer reliability analyses could not be performed for Psoas CSA and HU measurements because the current dataset included mean measurements rather than individual observer-specific values. Although measurements were independently performed and averaged by two experienced surgeons, future prospective studies should include formal reliability testing using intraclass correlation coefficients. Sixth, the “no new fracture” group was defined by the absence of a positive code, assuming full follow-up detection. Seventh, from this observational design, it cannot be determined whether the loss of significance of inflammatory markers in multivariate analysis reflects a true lack of independent contribution, a mediating effect via sarcopenia, or insufficient statistical power. Larger prospective multicenter studies including BMD/T-score data, formal measure reliability analysis, external validation, and mediating effect analysis are needed.

## 5. Conclusions

In a retrospective cohort of patients who underwent vertebroplasty or kyphoplasty due to osteoporotic spinal compression fractures, CT-derived Psoas muscle cross-sectional area and density were independently associated with the development of new spinal fractures. These imaging biomarkers demonstrated stronger independent predictive performance than systemic inflammatory indices in a multivariate model. The simple model showed good discrimination, adequate calibration, and stability on internal validation; however, the proposed thresholds, including the Psoas HU cutoff value of 20.50, are exploratory and require external validation. Prospective multicenter studies including BMD/T-score data, formal measurement reliability assessment, and longitudinal evaluation of muscle and inflammatory status are needed.

## Figures and Tables

**Table 1:** Comparison of parameters according to new fracture status (BH-adjusted).

Variable	Fracture (*n* = 49)	No Fracture (*n* = 117)	HLΔ	r_rb	Raw *p*	BH *p*	Significance
Age (years)	71.00 (65.00–78.00)	67.00 (59.00–74.00)	5.000	0.250	0.0112	0.0272	*
Delta VAS	3.00 (2.00–5.00)	−8.00 (−9.00 to −7.00)	12.000	1.000	<0.001	<0.001	***
Follow-up (mo)	12.00 (12.00–14.00)	12.00 (12.00–14.00)	0.000	0.042	0.630	0.669	
Psoas CSA (cm^2^)	3.95 (3.14–4.91)	5.43 (4.63–6.69)	−1.660	−0.622	<0.001	<0.001	***
Psoas HU	17.50 (14.00–19.50)	34.00 (30.00–37.50)	−16.500	−0.810	<0.001	<0.001	***
WBC	7.87 (6.20–9.28)	7.42 (6.17–8.95)	0.300	0.083	0.399	0.522	
Hemoglobin	11.90 (10.90–12.90)	12.50 (11.60–13.40)	−0.700	−0.238	0.016	0.033	*
Hematocrit	36.20 (33.40–38.90)	38.50 (35.10–41.20)	−2.000	−0.254	0.010	0.027	**
Platelet	265 (179–310)	259 (221–306)	−8.000	−0.058	0.558	0.632	
MPV (fL)	9.80 (8.80–10.50)	9.90 (9.00–10.60)	−0.100	−0.038	0.702	0.702	
Neutrophil	5.64 (4.38–6.53)	4.85 (3.52–6.10)	0.660	0.179	0.070	0.119	
Lymphocyte	1.51 (1.11–1.89)	1.95 (1.41–2.35)	−0.350	−0.303	0.002	0.009	**
Monocyte	0.52 (0.36–0.60)	0.52 (0.41–0.62)	−0.020	−0.074	0.455	0.552	
NLR	3.28 (2.24–5.39)	2.35 (1.55–3.77)	0.801	0.279	0.005	0.016	**
PLR	149.8 (122.8–214.6)	138.2 (105.1–188.6)	17.912	0.156	0.114	0.176	
LMR	3.22 (2.43–4.34)	3.71 (2.85–4.66)	−0.351	−0.124	0.210	0.298	
SII	843 (482–1376)	624 (385–982)	172.222	0.194	0.049	0.092	†

Values are given as median (IQR). HLΔ = Hodges–Lehmann median difference; r_rb = rank-biserial correlation. BH *p* = Benjamini–Hochberg adjusted *p*-value. † SII did not survive FDR correction. *, **, and *** = significant after BH correction.

**Table 2:** Univariate logistic regression for new fracture prediction.

Variable	OR	95% CI	*p*	Significance
Age	1.042	1.007–1.078	0.018	*
Gender (Female)	2.357	0.962–5.773	0.061	
Psoas CSA (cm^2^)	0.336	0.222–0.508	<0.001	***
Psoas HU	0.810	0.761–0.862	<0.001	***
Hemoglobin	0.749	0.600–0.934	0.010	*
Hematocrit	0.897	0.827–0.973	0.009	**
Neutrophil	1.143	0.975–1.341	0.100	
Lymphocyte	0.537	0.318–0.906	0.020	*
NLR	1.122	0.994–1.268	0.063	
PLR	1.002	0.999–1.005	0.207	
LMR	0.916	0.769–1.090	0.323	
SII	1.000	1.000–1.001	0.088	

*, ** and *** in the table indicate *p* < 0.05, *p* < 0.01 and *p* < 0.001, respectively.

**Table 3:** Parsimonious multivariate logistic regression model.

Variable	β	SE	z	*p*	OR	95% CI	Significance
Intercept	13.380	3.323	4.026	<0.001	-	-	***
Age	−0.052	0.030	−1.733	0.083	0.949	0.894–1.007	
Gender (Female)	−1.413	0.828	−1.707	0.088	0.244	0.048–1.233	
Psoas CSA	−0.908	0.287	−3.161	0.002	0.403	0.230–0.708	**
Psoas HU	−0.192	0.036	−5.374	<0.001	0.825	0.770–0.885	***

The regression equation was as follows: logit(P) = 13.380 − 0.052 × Age − 1.413 × Gender (Female) − 0.908 × Psoas CSA − 0.192 × Psoas HU. A forest plot, visualizing multivariate odds ratios with 95% confidence intervals, is presented in Supplementary Figure S1. ** and *** in the table indicate *p* < 0.01 and *p* < 0.001, respectively.

**Table 4:** ROC analysis: Sample-derived threshold values for predicting new fractures.

Variable	AUC	95% CI	Cut-Off	Sensitivity	Specificity
Psoas HU	0.905	0.844–0.955	20.50 HU	0.796	0.949
Psoas CSA	0.811	0.733–0.883	4.18 cm^2^	0.612	0.915
Lymphocyte	0.651	0.563–0.739	1.89 × 10^3^/µL	0.776	0.530
NLR	0.640	0.538–0.738	2.89	0.633	0.632
Age	0.625	0.536–0.718	61 years	0.918	0.333
Hemoglobin	0.619	0.522–0.710	12.20 g/dL	0.633	0.556
SII	0.597	0.493–0.699	760.8	0.592	0.650

Threshold values were determined by Youden’s J index in the study sample. These threshold are sample-derived, exploratory, have not been externally validated, and should be interpreted with caution.

## Data Availability

Datasets are available from the corresponding author upon reasonable request.

## Supplementary Materials

The following supporting information can be downloaded at: https://www.mdpi.com/article/10.3390/jcm15103677/s1, Figure S1: Forest plot of the parsimonious multivariate model. All VIF values < 2.3, EPV = 12.2. Red markers indicate *p* < 0.05; Figure S2: Calibration plot for the parsimonious model. Hosmer-Lemeshow *p* = 0.524. Point size is proportional to the decimal sample size. Lowess smoother is shown in blue.
