# Supplementary material for: Sarcopenia Versus Systemic Inflammation as Predictors of New Vertebral Fractures After Vertebroplasty or Kyphoplasty: A Retrospective Cohort Study
Source: J Clin Med. 2026 May 11;15(10):3677. doi: 10.3390/jcm15103677 (PMC13207675; doi:10.3390/jcm15103677)
Supplement: Supplementary file 1 [file jcm-15-03677-s001.zip › jcm-4271577-supplementary.pdf]

Supplementary Materials

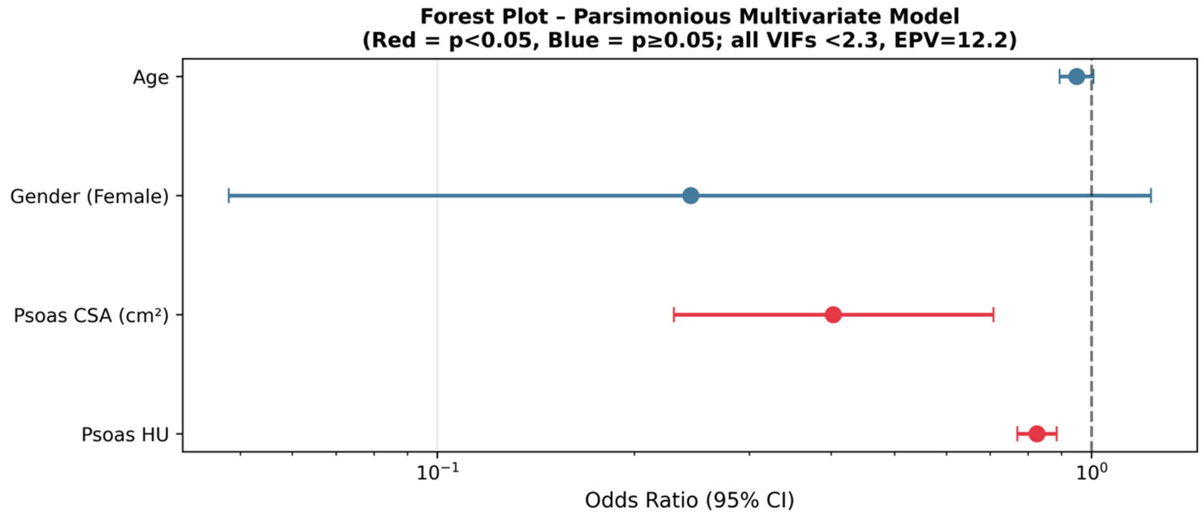

Supplementary Figure S1. Forest plot of the parsimonious multivariate model. All VIF values <2.3, EPV=12.2. Red markers indicate $p<0.05$.

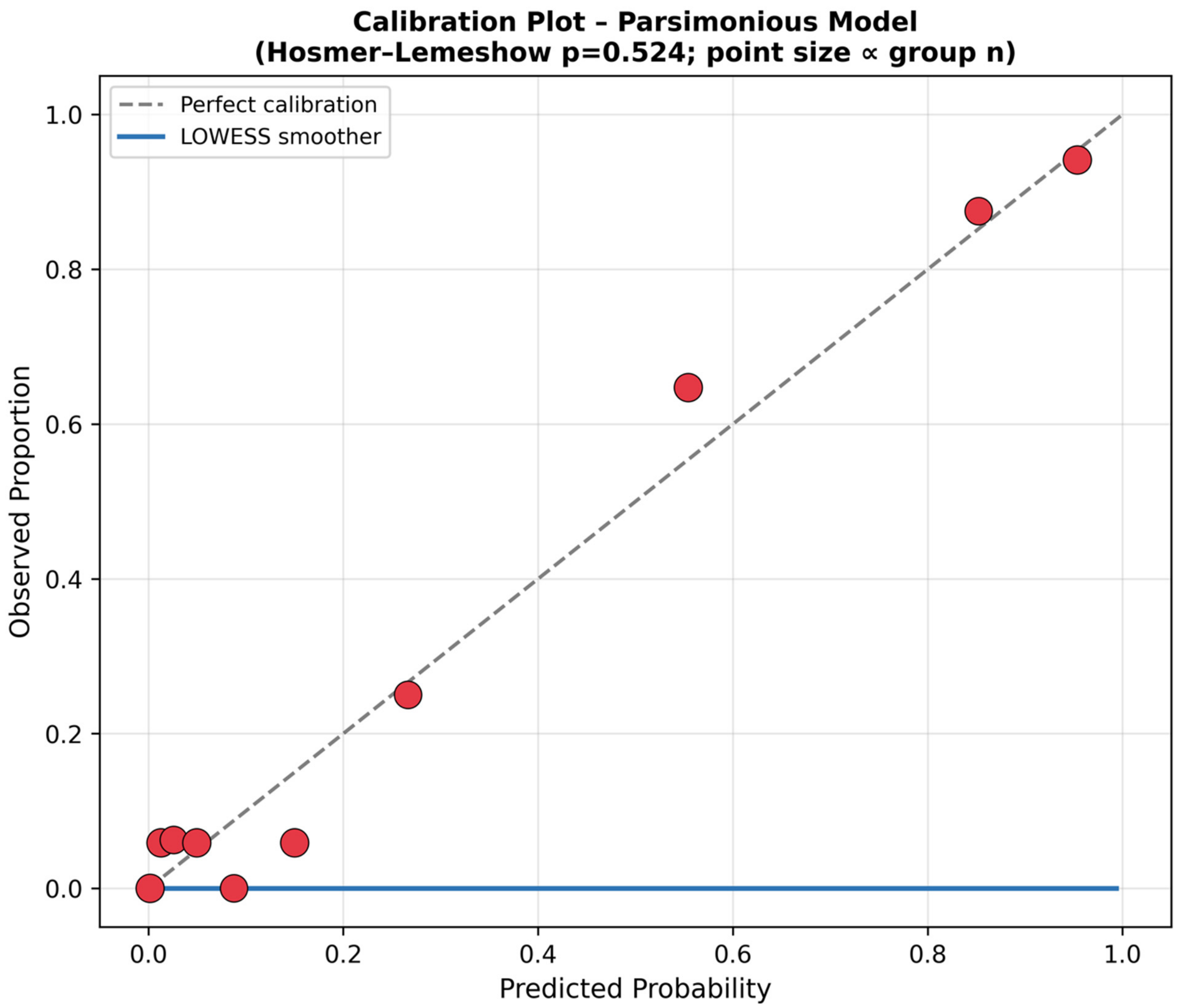

Supplementary Figure S2. Calibration plot for the parsimonious model. Hosmer-Lemeshow p=0.524. Point size is proportional to the decimal sample size. Lowess smoother is shown in blue.
